# Genomic Based Analysis of the Biocontrol Species *Trichoderma harzianum*: A Model Resource of Structurally Diverse Pharmaceuticals and Biopesticides

**DOI:** 10.3390/jof9090895

**Published:** 2023-08-31

**Authors:** Suhad A. A. Al-Salihi, Fabrizio Alberti

**Affiliations:** 1 Department of Applied Sciences, University of Technology, Baghdad 10066, Iraq; 2 School of Life Sciences, University of Warwick, Gibbet Hill Road, Coventry CV4 7AL, UK

**Keywords:** biocontrol, *Trichoderma*, secondary metabolites, genome mining, biosynthetic gene clusters

## Abstract

Fungi represents a rich repository of taxonomically restricted, yet chemically diverse, secondary metabolites that are synthesised via specific metabolic pathways. An enzyme’s specificity and biosynthetic gene clustering are the bottleneck of secondary metabolite evolution. *Trichoderma harzianum* M10 v1.0 produces many pharmaceutically important molecules; however, their specific biosynthetic pathways remain uncharacterised. Our genomic-based analysis of this species reveals the biosynthetic diversity of its specialised secondary metabolites, where over 50 BGCs were predicted, most of which were listed as polyketide-like compounds associated clusters. Gene annotation of the biosynthetic candidate genes predicted the production of many medically/industrially important compounds including enterobactin, gramicidin, lovastatin, HC-toxin, tyrocidine, equisetin, erythronolide, strobilurin, asperfuranone, cirtinine, protoilludene, germacrene, and epi-isozizaene. Revealing the biogenetic background of these natural molecules is a step forward towards the expansion of their chemical diversification via engineering their biosynthetic genes heterologously, and the identification of their role in the interaction between this fungus and its biotic/abiotic conditions as well as its role as bio-fungicide.

## 1. Introduction

The serious ecological and economical damage caused by some pathogenic microorganisms necessitates robust control strategies. The ubiquitous nature of fungi makes controlling their role in food stability especially challenging, preventing the use of traditional agronomic cultural control practices such as fallowing and long rotations. Due to their ability to parasitise other living systems, fungi can be sometimes devastating. There were several reports on the catastrophic effects of some fungal species on food stability, including the Bengal and the Irish famines. In addition to that, they can be pathogenic to humans [1].

Despite their beneficial uses, biological control usage has been proving challenging due to the potential interaction with invasive species, increasing crops’ varieties, pesticides competitions, random effects, as well as risk assessment policies [2,3]. Effective control strategies are therefore of urgent need to safeguard human social stability and survival [4].

The reported use of organisms as biological control agents can be dated to nearly a century ago [5]. During that time, it was estimated that annual crop losses in the US alone costs hundreds of billions of dollars [6], which was mainly associated to the interaction of many pathogenic fungal species with plants, as well as the rise in the population of insecticide resistance. However, many microbes, particularly fungi, have proven to be an effective tool in the management of pest attacks [7]. There are several factors that could influence the effectiveness of microbes as biological control agents including nutrient deficiency, familiar species of plant pathogens, as well as stress induced environment [8,9]. Generally, microbes’ products are preferred in terms of biological controls as they are cost effective products, can minimise the use of many harmful pesticides and they have a broad-spectrum efficiency [2,10]. A recent update on biological control sources, showed that around 31 fungal genera, most of which belong to the phylum Ascomycota, were used either directly or as active ingredient. The ability to produce specialised molecules with biological activities has promoted *Trichoderma* species as bio-fertilizers, bio-stimulants, and biological control agents [11]. Despite over 450 species of *Trichoderma* have been described so far, only a small ratio of this number is currently in use as biological control agents. *Trichoderma*’s ability in diminishing the growth of other inhabitants in their niche, is primarily due to their prolific metabolites profile. Apart from their role in organism’s fitness, they have been used successfully as both communication and defence agents with or against another microorganism in their environment [12,13,14].

Although many clinically valuable secondary metabolites have been reported from *Trichoderma*, only a handful of them have been investigated at the genetic level. However, the recent advancements in the genome analysis tools are promising, as more biosynthetic pathways and novel molecules can be characterised and modified [15]. Owing to their saprophytic lifestyle, *Trichoderma* fungi can survive a wide range of ecological conditions, including wood, soil, and animals. The first time *Trichoderma* was recognised as a biocontrol species was by [16], resulting in awareness of the side effects of the use of fumigants and chemical pesticides [17]. The ability to control the growth of a wide spectrum of plant pathogens has classified *Trichoderma* genus as one of the most efficient biocontrol species [18,19]. This ability is based on several strategies including the production of antagonistic materials and catalysing enzymes as well as nutrients competition. *Trichoderma* has also the ability to diminish the growth of other fungi in its environment (especially on plants) using its mycoparasitism mechanisms, surpassing other genera as bio-fungicides [8]. On the other hand, it can promote the growth of plants and enable them to tolerate unfavourable growth conditions such as drought and salinity [20]. The involvement of secondary metabolites produced by *Trichoderma* in their antagonistic activity against many microbes, has been evidenced in many reports [21,22].

The development of resistance to pesticides around the globe, has motivated the research community particularly the fungal scientists to combine efforts in combating this problem. A significant part of these efforts comes from genomic/computational analysis of many microbial genomes. The JGI community for instance, has recently launched the 1000 fungal genomes project, providing genome references for many scientists to further investigate these microorganisms’s capability as antagonists/biocontrols, pathogens, and decomposers [23]. Additionally, our previous work on genome analysis of basidiomycetes, proved that in-silico analysis of biosynthetic gene clusters can help in underlying the biogenetic production of several SMs, as well as in developing biological agents with novel antagonistic mechanisms [24,25]. We therefore selected the *Trichoderma* species, to underpin their metabolic pathways, as the genome of nearly nighty species of them are publicly available on JGI platform, of which we selected four isolates of the *Trichoderma harzianum* species (TR274, CBS226, T22, and M10 v1.0), owing to their ubiquitous activities in biocontrol and as prolific producer of biomedical agents, as well as their various metabolic pathway, especially the recently added strain *Trichoderma harzianum* M10 v1.0, which had a unique aromatic prenyltransferase (DMAT) within their biosynthetic pathways.

## 2. Materials and Methods

### 2.1. Trichoderma Species Genome Scan

The genome of twenty-three out of over eighty-six of the *Trichoderma* species [26,27,28,29,30,31,32,33,34,35,36,37,38,39,40,41,42,43,44,45,46,47,48], were manually scanned for potential novel secondary metabolites. All core enzymes potentially involved in the production of the three main types of SMs including polyketides (PKS), nonribosomal peptides (NRPS), terpene cyclase (TC), as well as the aromatic prenyltransferase-like enzyme dimethylallyltryptophan (DMAT) were investigated.

### 2.2. Validation of the M10 v1.0 Isolate and Annotation of the Core Enzymes of Its Secondary Metabolites (SMs)

Following the genomic scan of *Trichoderma* species, it appeared that amongst the *Trichoderma harzianum* species, the M10 v1.0 strain has the DMAT BGC. This gene cluster could potentially be responsible for unique features or functions in M10 v1.0 strain. Numerous genes have been used as biomarkers for the identification of *Trichoderma* species including those involved in secondary metabolism, mycoparasitism, and cell wall biosynthesis. Translation elongation factor (tef) for example, has shown to have high sequence variability among different *Trichoderma* species and can be used to differentiate closely related species. The chitinase gene (chi18-5) on the other hand, is involved in mycoparasitism, and can be used to characterise *Trichoderma* isolates with biocontrol potential, while endochitinase 1 gene (ech1), is used for the identification of *Trichoderma* species, especially those with high cellulase and xylanase activity such as *T. harzianum* Rifai. Additionally, two housekeeping genes; beta-tubulin (β-tubulin) and glyceraldehyde-3-phosphate dehydrogenase (gpdh), were also involved in species characterisation. We therefore performed a phylogenetic analysis of the above-mentioned conserved genes, to validate whether the genetic makeup of M10 v1.0 strain was distinct from other strains of *Trichoderma harzianum*, as well as to reconcile the inconsistent classification of *T. harzianum* strains [49,50,51], and possibly identify any unique traits that could set it apart from the other strains. The evolutionary relationship between the strains was inferred by using the Maximum Likelihood method and Whelan and Goldman model. The tree with the highest log likelihood (−5181.56) is presented and shows the percentage of trees in which the associated taxa clustered together. Initial tree (s) for the heuristic search were obtained automatically by applying the Maximum Parsimony method. A discrete Gamma distribution was used to model evolutionary rate differences among sites (5 categories (+*G*, parameter = 200.0000)). This analysis involved 20 amino acid sequences. All positions with less than 95% site coverage were eliminated, i.e., fewer than 5% alignment gaps, missing data, and ambiguous bases were allowed at any position (partial deletion option). There was a total of 369 positions in the final dataset. The evolutionary analyses were performed using MEGA11 [52]. This was followed with multiple databases analysis using JGI [23] alongside antiSMASH [53] and MIBiG [54] platforms, to analyse the potential core enzymes involved in its metabolic pathway. The predicted genes were subsequently evaluated through BlastP search against NCBI database for potential homologous in other species.

### 2.3. Secondary Metabolites Phylogenetic Analysis

To determine any potential evolutionary connections that might exist between the predicted core enzymes and other experimentally characterised secondary metabolites, we constructed a phylogenetic tree for each type of the predicted enzymes using the software Mega11 [52]. We used the Maximum Likelihood method and the Whelan Goldman model. To provide insight into the evolutionary history and relationships of the genes selected from M10 v1.0 isolate and other experimentally validated biosynthetic genes. The generated trees illustrate the relationships between the genes and have the highest log likelihood values (−30,402.32, −29,486.13, −17,158.06 for NRPS, PKS, and TC trees, respectively) out of all the trees produced during the analysis. The initial trees used for the analysis were obtained through the Neighbor-Join and BioNJ algorithms, which estimated pairwise distances using the JIT model, and then selected the topology with the best log likelihood value. A discrete Gamma distribution with a parameter value of 11.8827, 3.2345, and 15.0423 (for NRPS, PKS, and TC trees, respectively) and 5 categories were used to model evolutionary rate differences among sites. The trees are drawn to scale, with branch lengths measured in the number of substitutions per site. The study involved 54, 61, and 36 amino acid sequences for NRPS, PKS, and TC trees, respectively), and all positions with less than 95% site coverage were removed from the analysis. The final dataset contained a total of 285, 344, and 205 positions for NRPS, PKS, and TC trees, respectively. For tree reliability test we selected the bootstrap method with an average of 100 replicates. All evolutionary analyses were performed using Mega11 [52].

### 2.4. Annotation of the Predicted Biosynthetic Gene Clusters

Each predicted core enzyme was individually analysed through BlastP search against the JGI database to localise its association with biosynthetic gene clusters on *Trichoderma*’s M10 v1.0 genome and annotated through homology search against the NCBI database. Whether the annotated core enzymes are part of a metabolite associated biosynthetic gene cluster was putatively determined by examining the respective genomic scaffold of *Trichoderma*’s genome. Cluster layouts were drawn using MS Word and PowerPoint.

## 3. Results

### 3.1. Genome Scan

**Figure 1:**
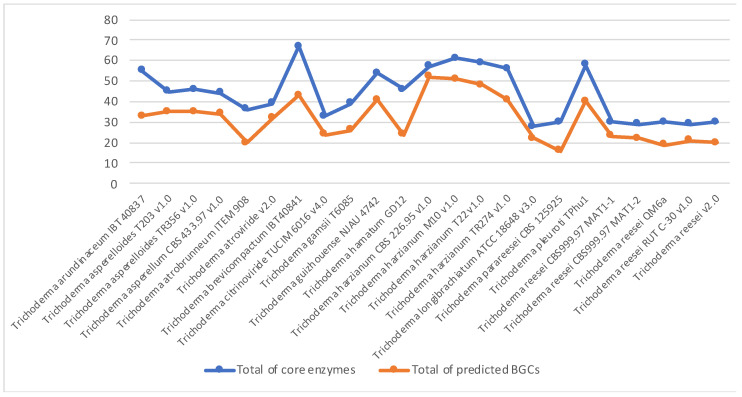
*Trichoderma* species SMs core enzymes and their associated BGCs.

**Figure 2:**
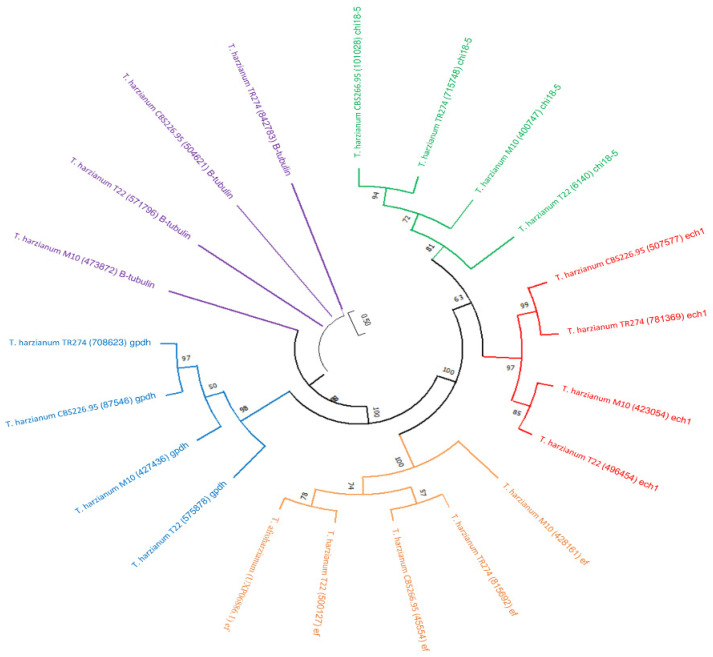
Maximum likelihood tree of five conserved genes (chitinase gene {chi18-5}, endochitinase1 {ech1}, β-tubulin, glyceraldehyde-3-phosphate dehydrogenase {gpdh}, and translation elongation factor {tef} of *T. harzianum* M10 v1.0, *T. harzianum* CBS226.95, *T. harzianum* TR274, *T. harzianum* T22, and *T. afroharzianum*. Nodes labels indicate species taxon-protein ID-gene function.

Several biologically important compounds have been described from *Trichoderma* ssp. indicating that this genus is capable of producing a wide range of secondary metabolites; yet, very few associated BGCs (if present) have been characterised from this genus [55]. The genomic data of twenty-three out of approximately ninety *Trichoderma* species were available on JGI, which we scanned to select the most promising profile. Manual investigation was carried out using the JGI platform. We navigated each genome by locating each predicted core enzyme of three main SMs (PKS, NRPS, and TC) as well as the DMAT, to their BGC or surrounding genes, resulting in many pseudo/duplicated BGCs (Figure 1 and Table S1 in the supplementary information). Furthermore, the phylogenetic analysis of five conserved genes of the four selected *T. harzianum* isolates, allowed for a better understanding of the evolutionary relationships between these isolates, and further confirmed that the M10 v1.0 isolate belongs to the *T. harzianum* family, as high sequence similarity was observed between its conserved genes and the other *T. harzianum* isolates (Figure 2).

### 3.2. Trichoderma Harzianum Genomes Comparison

*Trichoderma harzianum* species are famously known for their ubiquitous activities as biocontrol and prolific producers of biomedical agents, and to determine the biological role of any potential SM biosynthetic genes, the selected four *Trichoderma harzianum* genomes were searched carefully, through a subsequent genome investigation on antiSMASH. We first downloaded the whole genome of each isolate from the JGI portal, and then submitted it to antiSMASH, where a total of 190 BGCs including 57 NRPs, 74 PKS, 24 TC, and 35 hybrid NRPs-PKS were detected. The antiSMASH prediction also included the “most similar cluster” prediction, where sequences homologous (33–100%) to other antibiotics BGCs could be obtained, including Metachelin A-C, Ochratoxin, Tricholignan, Dichlorodiapothin, Trichoxide, Harzianopyridone, Calvaric acid, Hariphilone, Squalestatin, Depudecin, Trichobrasilenol, Choline, Peramine and Lucilactaene (Table 1). Whereas a total of 241 BGCs including 22 hybrid, 96 NRPs, 99 PKS, and 24 TC clusters were predicted on the JGI website. In addition to that, the genome of the *T. harzianum* M10 v1.0, included a potential novel DMAT-BGC, we therefore, selected this genome for further BGCs annotation.

#### 3.2.1. NRPS and NRPS-Like Phylogenetic Analysis and Biosynthetic Gene Clusters Annotation

**Figure 3:**
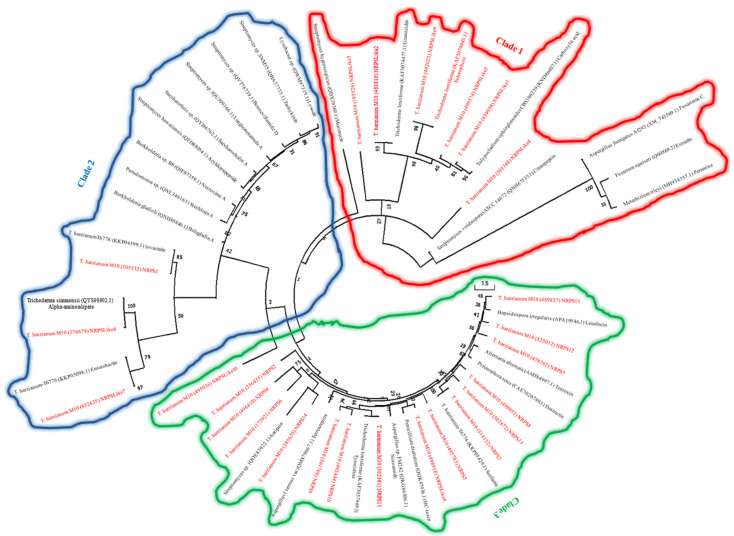
Maximum likelihood tree of the core NRPS/NRPS-like protein sequences of *T. harzianum* M10 v1.0 and other experimentally described NRPSs of different microbial species. Nodes labels indicate species taxon-protein ID-chemical. *T. harzianum* M10 predicted proteins are in red.

In our study, 19 NRPS/NRPS-like backbone enzymes were predicted in the genome of the examined *T. harzianum* M10 v1.0. To reveal any potential relationship between these NRPS backbones and previously characterised NRPS-like antibiotic biosynthetic gene clusters, including those being described from other *Trichoderma* ssp., a phylogenetic tree based on a collection of NRPS enzymes from the selected *Trichoderma* in addition to other randomly selected NRPSs from the MIBiG database that represents part of experimentally characterised biologically active NRPSs, was constructed using Maximum Likelihood. The resulted tree consisted of three major clades, and revealed the following homology of sequences between the *Trichoderma* NRPS/NRPS-like core enzymes and other previously characterised NRPS antibiotic related enzymes. Although, Tricho-NRPS-like1, 2, 3, 4, 5, and 9, have clustered with several antibiotic enzymes in clade 1, Tricho-NRPS-like 1, Tricho-NRPS-like 2, and NRPS-like 9, appeared to be closer to carboxylic, gramicidin, and siderphore than Tricho-NRPS-like3, Tricho-NRPS-like5 and Tricho-NRPS-like4, respectively. In clade 2, on the other hand, the three NRPS enzymes including NRPS1, NRPS-like7, and NRPS-like8, were closely clustered with enzymes responsible for the production of lovastatin, enterobactin, and alpha-aminoadipate one to one. The remaining fifteen NRPSs were all clustered in clade 3, where Tricho-NRPS11, 3, 6, 15, and 14 seemed to have homologous sequences to several medically important NRPS enzymes such as leualacin, surfactin, HC-toxin, tyrocidine, and antipain, respectively, Figure 3.

**Figure 4:**
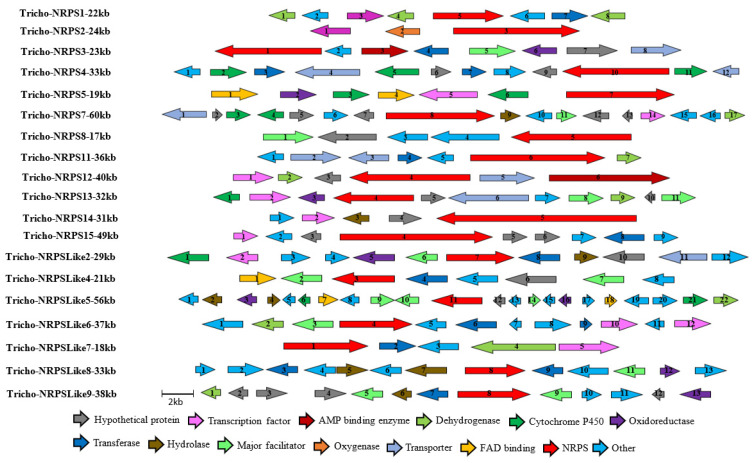
Organization of the genetic structure of the predicted non-ribosomal peptide BGCs of *Trichoderma harzianum* M10 v1.0 Sizes and directions of arrows represent different genes sizes and their 5′-3′ direction. Full description of gene function is provided in Tables S2–S20 in the supplementary information.

Anticipating that some of the predicted NRPSs might be associated with the biosynthesis of uncharacterised SMs, the genomic regions surrounding each backbone enzyme were mined for a possible BGC. Out of the 25 predicted NRPS/NRPS-like enzymes, 19 were found to be organised within a BGC, as shown in Figure 4.

#### 3.2.2. PKS and PKS-like Phylogenetic Analysis and Biosynthetic Gene Clusters Annotation

**Figure 5:**
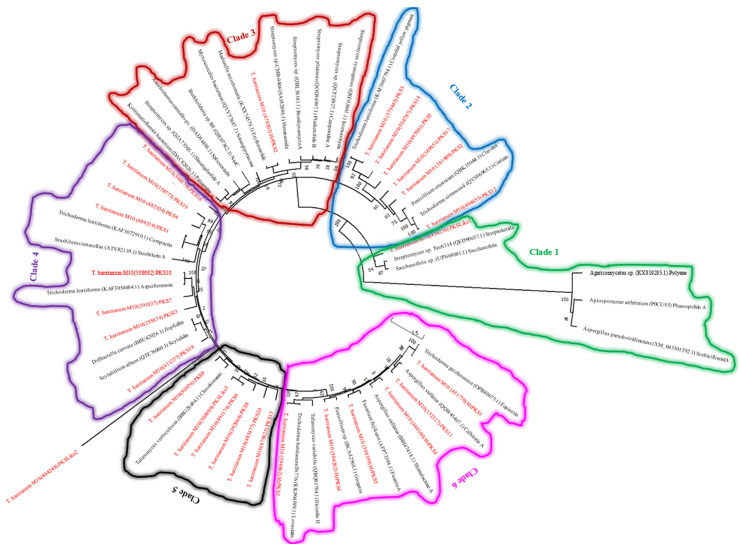
Maximum likelihood tree of the core PKS/PKS-like of *T. harzianum* M10 v1.0 and other experimentally described NRPS protein sequences of different microbial species Nodes labels indicate species taxon-protein ID-chemical. *T. harzianum* M10 predicted proteins are in red.

In a similar manner to the NRPSs, the predicted *Trichoderma* PKS biosynthetic genes were aligned with other microbial PKSs selected from the MIBiG database, and resulted in several examples of sequence homology that are clustered in six clades in a one maximum likelihood tree, as shown in Figure 4. The Tricho-PKSLike1, for example, formed clade one with the streptoketide, saccharothrix, polyene, phaeospelide, and sorbicillinoid antibiotic enzymes. Clade 2 involved more Tricho-PKS enzymes, such as Tricho-PKS3, Tricho-PKS14, Tricho-PKS5, Tricho-PKS17, Tricho-PKS2, and Tricho-PKS12, of which only Tricho-PKS3 and 12 showed high sequence homology with conidial and citrinin antibiotic enzymes, respectively. Clade 3 mostly consisted of antibiotic enzymes of other microbes with one Tricho-PKS (PKS3), which shared homologous sequences with the erythronolide antibiotic gene cluster. On the other hand, clade 4 gathered enzymes of three groups, one included four Tricho-PKS enzymes, of which Tricho-PKS1 was the closest to the antibiotic compactin, second contained three PKS enzymes, including the Tricho-PKS19 which seemed to be closely related to the asperfuranone antibiotic, and in the last group, the Tricho-PKS18 shared homologous sequences with both zopfiellin and scytalidin antibiotics. Clade five was more likely a Tricho-PKS enzymes clade, as it gathered six enzymes of *Trichoderma* with the enzyme responsible for one antibiotic that is chrodrimanin. In contrast, clade six, demonstrated more diversity, in terms of sequence homology between the Tricho-PKS enzymes and the selected antibiotic enzymes, such as the sequence similarity between Tricho-HrPKS1 and equisetin, Tricho-PKS11 and calbistrin, Tricho-HrPKS4 and shimalactone, Tricho-HrPKS5 and fusarin, Tricho-HrPKS6 and gregatin, and finally Tricho-HrPKS3 with lovastatin, as shown in Figure 5.

**Figure 6:**
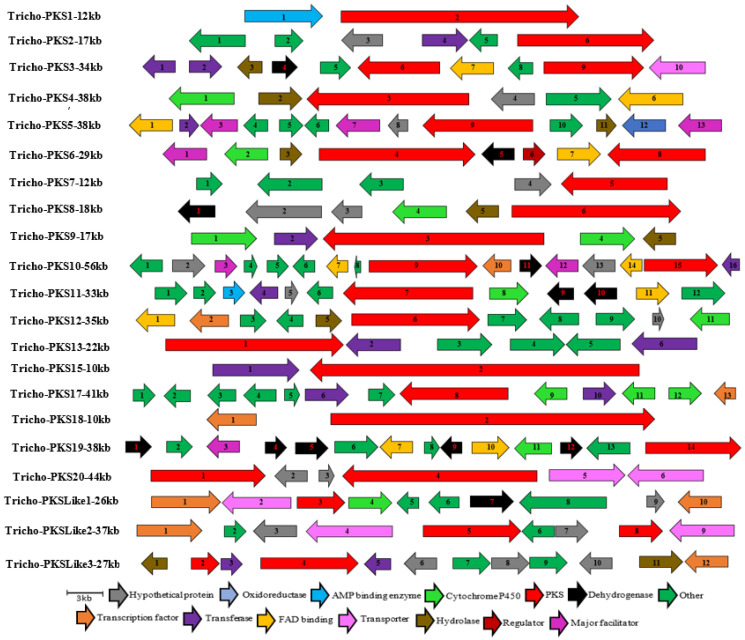
Organization of the genetic structure of the predicted polyketide synthase (PKS/PKS-like) BGCs of *Trichoderma harzianum* M10 v1.0. Sizes and directions of arrows represent different gene sizes and their 5′-3′ direction. Full description of gene function is provided in Tables S21–S41 in the supplementary information.

**Figure 7:**
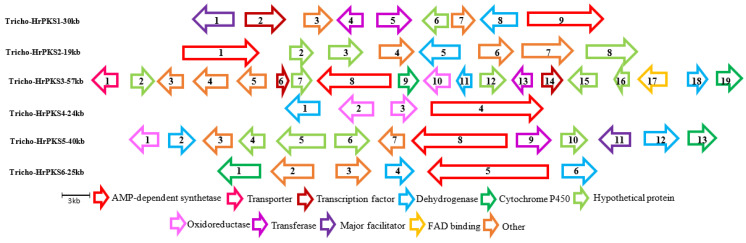
Organization of the genetic structure of the predicted hybrid polyketide synthase (HrPKS) BGCs of *Trichoderma harzianum* M10 v1.0. Sizes and directions of arrows represent different genes sizes and their 5′-3′ direction. Full description of gene function is provided in Tables S42–S47 in the supplementary information.

Given the potential importance of the other biosynthetic genes, we further annotated the genes surrounding the PKS core enzymes, and 10 putative BGCs were predicted. Looking at those clusters, it can be hypothesized that these clusters may encode the enzymes that could be responsible for the production of many medically relevant compounds, as shown in Figure 6 and Figure 7.

#### 3.2.3. Terpene Cyclase Phylogenetic Analysis and Biosynthetic Gene Clusters Annotation

**Figure 8:**
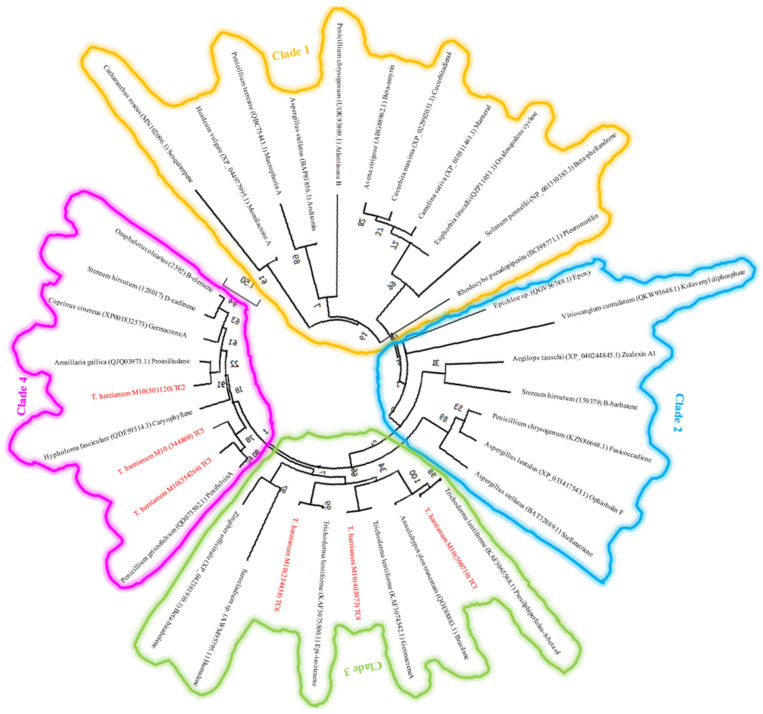
Maximum likelihood tree of the core terpene cyclase (TC) of the *T. harzianum* M10 v1.0 and other experimentally described TC protein sequences of different microbial species. Nodes labels indicate Species taxon-protein ID-chemical. *T. harzianum* M10 predicted proteins are in red.

**Figure 9:**
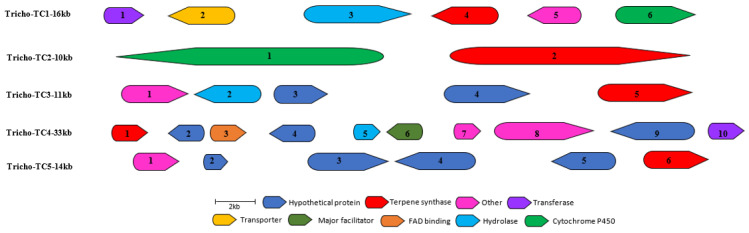
Organization of the genetic structure of the predicted terpene cyclase (TC) BGCs of *Trichoderma harzianum* M10 v1.0. Sizes and directions of arrows represent different gene sizes and their 5′-3′ direction. Full description of gene function is provided in Tables S48–S52 in the supplementary information.

**Figure 10:**
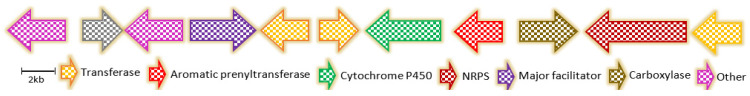
Organization of the genetic structure of the predicted dimethylallyltryptophan (DMAT) BGC of *Trichoderma harzianum* M10 v1.0. Sizes and directions of arrows represent different gene sizes and their 5′-3′ direction. Full description of gene function is provided in Table S53 in the supplementary information.

In addition to the prediction of the NRPS and the PKS core enzymes, we also predicted core enzymes for the production of terpene-like molecules, for which a maximum likelihood tree was also constructed using a combination of protein sequences of other microbial antibiotics selected from the MIBiG database, and unearthed highly closely related core enzymes for the production of many SMs and bioactive molecules, including sequence similarity between TC1 and presilphiperfolan-8-beta-ol from other *Trichoderma* sp., Tricho-TC2 with protoilludine, Tricho-TC3 and Tricho-TC5 with penifulvin, and Tricho-TC4 with germacrene A. Five of the six identified terpene cyclases were co-localised with other biosynthetic genes including cytochrome P450s, FAD oxidoreductases, different substrate transporters as well as several functionally unknown genes which were annotated as hypothetical proteins, as shown in Figure 8 and Figure 9. Additionally, an aromatic prenyltransferase-like enzyme dimethylallyltryptophan biosynthetic gene cluster (DMAT-BGC) was also predicted, as shown in Figure 10.

### 3.3. Clinker Investigation

#### 3.3.1. Core Enzymes Cblaster

**Figure 11:**
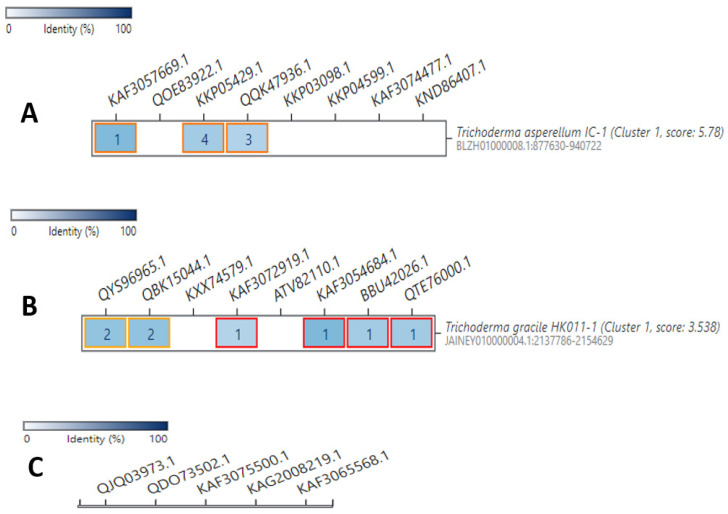
Cblaster analysis of three types of SMs enzymes that had high percentage matches with the *T. harzianum* M10 v1.0 SMs enzymes in our phylogenetic analysis. (**A**) Eight NRPS genes of *T. harzianum* were used as query, three of which had homologous sequence with *T. asperellum*. (**B**) Eight PKS genes of *T. harzianum* were used as query, five of which had homologous sequence with *T. gracile*. (**C**) Five TC genes of *T. harzianum* were used as query, none of which had sequences similarity with other organisms on NCBI database. A darker shade of blue denotes a higher percentage identity of the query in the output cluster, while the number within each box, resembles the counts of hits for a specific query sequence in the co-localized region. Orange and red borders indicate that similar genes found in multiple clusters.

To further confirm our phylogenetic results, we performed cblaster [56]. Analysis for the antibiotic enzymes that have shown high sequence similarity with our selected *Trichoderma* SM enzymes. A set of enzymes sequences/accession number of each group of the investigated SM were provided to cblaster to search the local/remote and the NCBI database for homologous core enzymes in other potential *Trichoderma* species that might be missed out during our genomic analysis. For the NRPS enzymes set, for example, we provided the genomic context of the following genes: tyrocidine (KAF3057669.1), antipain (QOE83922.1), surfactin (KKP05429.1), HC-toxin (QQK47936.1), enterobactin (KKP03098.1), lovastatin (KKP04599.1), gramicidin (KAF3074477.1), and carboxylic acid (KND86407.1), and the PKS enzymes provided set involved citrinin (QYS96965.1), clavatol (QBK15044.1), erythronolide (KXX74579.1), compactin (KAF3072919.1), strobilurin A (ATV82110.1), asperfuranone (KAF3054684.1), zopfiellin (BBU42026.1), scytalidin (QTE76000.1), while for the TC enzymes, we inputted protoilludane (QJQ03973.1), penifulvin A (QDO73502.1), epi-isozizaene (KAF3075500.1), germacrene A (KAG2008219.1), presilphiperfolan-8-beta-ol (KAF3065568.1) proteins as query. Among all searched *Trichoderma* species, only *Trichoderma asperellum* demonstrated potential similarity (around 30%) with three of the eight investigated NRPS enzymes. Likewise, our query of the selected PKS genes, resulted in sequences similarity (less than 30%) with six of the eight examined genes. In contrast, none of the investigated TC enzymes resulted in sequence matches (Figure 11).

#### 3.3.2. Biosynthetic Gene Clusters Cblaster

The outcome of antiSMASH analysis of the four selected *Trichoderma harzianum* isolates suggested the presence of several BGCs for pharmaceutically relevant compounds (Table 1), some of which might be uniquely present in the *harzianum* species, and to further confirm this, we carried out cblaster analysis for the predicted BGCs on antiSMASH, of which we selected the clusters that had their core set of genes (essential for compound production) experimentally characterised, and had the highest identity percentage with the investigated *Trichoderma* core enzymes. Selected clusters included ochratoxin, clavaric acid, harziphilon, tricholignan, choline, and peramin. Each cluster was further investigated individually through cblaster against local/remote genome database. The outcome of such analysis has further confirmed the antiSMASH prediction, and suggested that two of these BGCs, namely clavaric acid and choline, are present only in the *harzianum* species among other *Trichoderma* species, as shown in Table 2 (see repository on https://github.com/Suhadbio (accessed on 11 May 2023) for details of the cblaster analysis using the ochratoxin, clavaric acid, harziphilon, tricholignan, choline, and peramin gene clusters as queries, respectively).

## 4. Discussion

The fungal species *Trichoderma harzianum* is widely known as a biocontrol agent and biofertilizer, which utilises its SMs to interact with its ecosystem. Unfortunately, many of those SMs are not produced under laboratory conditions, as their associated gene clusters are silent [55]. We therefore performed a genomic-based sequence analysis to predict candidate genes for potential bioactive molecules in four selected *Trichoderma harzianum* isolates via means of JGI [38,57], antiSMASH, NCBI blastP, and cblaster. Based on the obtained results in Table 1, it is evident that some gene clusters responsible for the biosynthesis of molecules such as trichoxide, harzianopyridone, tricholignan, and cholin are conserved across different *T. harzianum* isolates. This suggest that these compounds may have important biological and ecological roles in these species. However, our results also revealed notable diversity and complexity in the chemical profiles of *T. harzianum* isolates, with isolate-specific molecules such as ochratoxin and lucilactaene predicted in the M10 isolate. This highlights again the potential of *T. harzianum* to produce a broad range of bioactive molecules that could be useful in various industries including agriculture and medicine [9,55,58].

Following that, we performed manual sequence alignment to predict the homology and variations between the predicted core enzymes and other previously characterised bioactive SMs. This has revealed high sequence similarity with more antibiotic core enzymes of the three types of the SMs. The NRPS antibiotics that had high percentage of sequences homology with our NRPS core enzymes included leualacin, surfactin, HC-toxin, tyrocidine, antipain, enterobactin, lovastatin, gramicidin, siderphore, and carboxylic acid while the PKS antibiotics highest matches were citrinin, clavatol, erythronotide, sorangipyranone, compactin, strobilurin, asperfuranone, zopfiellin, scytalidin, lovastatin, illicicolin, gregatin, fusarin, and equisetin. Finally, the TC antibiotics matches were protoilludine, penifulvin, presilphiperfolan-8-beta-ol, germacrene, and epi-isozizaene. While other *Trichoderma* sp. are known for the production of a few of the above predicted molecules, such as squalestatin, naphthopyrone, dimethylcoprogen, and clavaric acid [59], interestingly, our sequence analysis shed light on many more potential bioactive molecules that are most likely first reported in *Trichoderma harzianum* M10 v1.0.

Generally, the non-ribosomal peptide synthetases are large enzymes that are associated with the production of cyclic peptide natural products. Typically, they consist of a range of modules, each of which contains the three standard domains structure of NRPS enzymes; the adenylation (A) domain, the condensation (C) domain, the thiolation (T) domain. However, this is not always the case, as some modules may lack a condensation domain while others may include extra domains in their structure, such as an epimerization (E) domain, N-methylation (M) domain, and a heterocyclization (Cy) domain. These extra domains are usually associated with the modification of the amino acid substrate complex, and more often influence the bioactivity spectrum of such enzymes [60].

Some of the above identified/described SM BCGs are involved in the synthesis of toxins, while others are responsible for the production of pharmaceutically important molecules. To begin with the molecule antipain for example, is medically known as a protease inhibitor, and is produced by many bacterial species, and a BGC was recently identified from the producer species *Streptomyces* sp. ID38640 that is responsible for making it [61]. The cinnapeptin was recently added to the depsipeptide family alongside other depsipeptide molecules including atratumycin, skyllamycin, and kitacinnamycin [62,63,64]. It has a rare cyclization pattern that is shared with its related atratumycin. In terms of its synthesis, cinnapeptin is usually synthesized by the Phe ammonia lyase, which is attributed to type II PKSs, particularly to non-proteinogenic amino acids, suggesting a NRPS-T2PKS participation in such biosynthesis; however, a detailed pathway is yet to be characterised [65]. The stechlisins are cyclic lipoproteins that are structurally related isomers and have been characterised from *Pseudomonas* sp. FhG100052 recently. The structure diversity in stechlisins is due to the positional interchange of either the aliphatic or the acidic residues. In addition to that, these amino acids represent the substrate flexible family, meaning that their A domains have similar substrate specificities [64,66] and that they are named after their peptide positions. It can be therefore postulated that stechlisins associated enzymes are able to produce many other structurally diverse compounds that could have pharmaceutical properties [67]. Tentoxin, is a member of the cyclic tetrapeptide family, that has the ability to block the F1-ATPase in many plant chloroplasts, classifying it as a potential herbicide [68]. What is special about these cyclic peptides, is the presence of several unusual non-proteinogenic residues, to which many pharmaceutically important molecules including cephalosporin, penicillin, vancomycin, and gramicidin belong [69]. Terreazepine represents an unusual compound produced by a duplicated neo-functionalized indoleamine diozyenase that is only present in *Aspergillus terreus*. Its biosynthesis includes an unusual cyclization pattern of the precursor kynurenine [70]. HC-toxin, is capable of inhibiting the histone deacetylases of the RPD3, enabling its producer to infect a variety of maize plant species, and participate in the selective pressure on many grasses’ evolution. HC-toxin synthase is a tetra module non-ribosomal peptide synthetase with one epimerase domain that has been characterised in the pathogenic fungus *Cochliobolus carbonum* [71]. Destruxin synthase has only recently been characterised through gene targeted disruption in the pathogenic fungus *Metarhizium robertsii* [72], despite destruxin having been known for a long time. It was found that the non-ribosomal peptide synnthetase responsible for destruxin production consists of six adenylation domains, of which two are selective in terms of amino acids they bind to during the biosynthesis process. So far, six groups of destruxins have been characterised, most of which are produced by the species *Metarhizium anisopliae*. Destruxin compounds have insecticidal effects, they can cause paralysis and intestinal construction as well as block the Ca^2+^ channel of the ATPase [73,74]. Both gramicidin and tyrocidine are cyclic decapeptide compounds that are synthesized by three mega-synthetases including a non-ribosomal peptide via the thiol template biosynthetic pathway. These compounds are pharmaceutically important as they are capable of penetrating the lipid layer of many Gram-positive bacteria including antibiotic resistant ones, and ultimately disrupt their membrane [75,76]. Leualacin is chemically recognised for its unique cyclic depsipeptide structure, and it was first characterised from the fungus *Hapsidospora irregularis*. In terms of its pharmacological role, leualacin is known for its effectiveness in blocking calcium channels [77]. Finally, enterobactin, made by both *Salmonella* and *E. coli*, is naturally synthesised from the precursor chorismic acid via an NRPS in a two-step reaction [78,79].

As for the PKS predicted matches in this study, they included equisetin, a broadly known toxic substrate, that is mainly derived from the polyketide derivative decalin and the amino acid derivative tetramic acid, and has backbone similarity to other distantly related molecules such as lovastatin. This compound has been used as a source for the development of pharmaceutical and agrochemical materials for many years; yet, only recently its biosynthetic gene cluster has been identified [80,81]. Lovastatin was first isolated from *Aspergillus terreus* in 1978, and later in *Monascus ruber* in 1979 [82]. The nonaketide enzyme that encodes lovastatin synthase consists of six domains including KS, AT, DH, MT, KR, inactive ER, and ACP domains. Lovastatin’s therapeutical uses include the treatment of hypercholesterolemia, as it inhibits the enzyme responsible for catalysing the rate limiting step of the biosynthesis of cholesterol. Another known use of lovastatin is its involvement in the semi-synthesis of the drug simvastatin [83]. On the other hand, surfactin is an antimicrobial molecule that actively alters membrane integrity of microbes. It is mainly synthesised by a heptapeptide type of PKS which is distinguished with its LLDLLDL chiral sequences that are linked to a hydroxy fatty acid by a lactone bond [84]. Additionally, erythronolide is a member of the type I polyketide synthase enzyme family, and capable of catalysing reactions involving propionyl-CoA and malonyl-CoA substrates. It is generally considered to be a transferase enzyme, and is found to be responsible for the synthesis of the building blocks of erythromycin derivatives. Fusarin-A is naturally produced by different species of the genus *Fusarium*, and its biosynthesis is largely induced by nitrogen availability. The enzyme responsible for the production of this compound consists of 10 protein domains, 4 of which are characteristic of NRPS domains, making it the largest enzyme member of its family. What is unusual about these NRPS domains is that they are specifically involved in the synthesis of the pyrrolidone ring of fusarin, and their sequences could be used for the identification of other fusarin-producing species, particularly those in relation with plants [85]. Furthermore, the asperfuranone, which is an unusual PK molecule that is produced by the fungus *Aspergillus nidulans* via the participation of two PKSs of the non-reducing type [86]. Finally, citrinin is a polyketide mycotoxin that has potential carcinogenic, nephrotoxic and hepatotoxic activities, and is produced by several species including *Aspergillus* sp., *Monascus* sp., and *Penicillium citrinum* [87].

In terms of TC antibiotics matches, and as noted from the phylogenetic tree, there were three antibiotic core enzymes that demonstrated sequence homology to our TC core enzymes, including the presilphiperfolan-8-ol, a tricyclic alcohol terpen precursor. It is found that the cyclase responsible for the production of the sesquiterpene botrydial, the phytotoxin that causes the grey mould disease in a wide range of plants, is one of the genes involved in the synthesis of presilphiperfolan-8-ol [88]. Germacrene A is a sesquiterpene-like compound that was first characterised from *Eunicea mammosa* in the 1970s. Biologically, it is synthesised from the terpene precursor farnesyl diphosphate (FPP) via germacrene synthase following a 1, 10-cyclisation pattern [24,89]. Finally, epi-isozizaene is a bacterial sesquiterpene that is structurally related to pentalenen. Its biosynthetic enzyme, epi-isozizaene cyclase, was first characterised from *Streptomyces coelicolor* A3(2) [90]. Epi-isozizaene cyclase is a promiscuous enzyme that can produce several end products, with one being the major. Our predicted core biosynthetic genes were often co-localized on contigs with other modifying genes including FAD-binding proteins, short chain dehydrogenase, cytochrome P450, substrate transporters, aldo-keto reductase, O-methyltransferase, and regulatory factors. Localising similar genes in *Trichoderma*, will likely help in the identification of overlooked potential pharma-agro related molecules [87].

## 5. Conclusions

Based on chemical and analytical studies, the *Trichoderma* genus is a prolific producer of SMs, as more than 300 molecules have been characterised and far more than that number was estimated to be produced over the years from different species of this genus. However, the specific biological activity and the biogenetic origin of the identified molecules remained largely unknown, as only a handful of them including gliotoxin and several trichothecenes have been experimentally investigated. However, with the rapid advances in computational genomic analysis, and more and more genomes being sequenced, one can hope that this scenario can be changed and that more metabolite pathways can be elucidated as well as more novel molecules can be discovered and modified. Our genome annotation together with the phylogenetic and cblaster analysis, further confirm the high potential of *T. harzianum* species as untapped source of pharmaceutically relevant and agrochemical molecules.

## Figures and Tables

**Table 1:** Homologous sequences percentage of the four selected *Trichoderma harzianum* predicted BGCs with other characterised antibiotic BGCs on antiSMASH.

Most Similar BGC on antiSMASH	BGC Type	*Trichoderma harzianum* CBS 226.95 v1.0 (Triha1)	*Trichoderma harzianum* T22 v1.0 (TriharT22)	*Trichoderma harzianum* TR274 v1.0 (Trihar1)	*Trichoderma harzianum* M10 v1.0 (TriharM10)
Metachelin A-C	NRPS	62%	75%	62%	62%
Ochratoxin	NRPS-PKS	X	X	X	100%
Dichlorodiapothin	PKS	50%	50%	50%	33%
Trichoxide	PKS	75%	83%	75%	83%
Harzianopyridone	NRPS-PKS	60%	60%	60%	70%
Clavaric acid	Terpene	100%	X	100%	100%
Harziphilone	PKS	80%	80%	80%	80%
Squalestatin	Terpene	40%	40%	X	40%
Depudecin	PKS	33%	33%	33%	33%
Trichobrasilenol	Terpene	60%	60%	60%	X
Tricholignan	PKS	100%	100%	100%	100%
Choline	NRPS	100%	100%	100%	100%
Peramine	NRPS	X	100%	X	100%
Lucilactaene	PKS	X	X	X	38%

**Table 2:** Outcome of cblaster analysis of six BGCs that are identified within antiSMASH as homologous clusters of the *T. harzianum* M10 v1.0.

BGC of Interest	No. of Organisms Searched	No. of Genomic Scaffold Searched	No. of Scaffolds Identified with the BGC	No. of *Trichoderma* Species with Similar BGC
Ochratoxin	416	566	573	18
Clavaric acid	513	568	559	None
Harziphilon	631	2971	735	9
Tricholignan	93	390	96	7
Choline	1088	1134	1133	None
Peramine	346	628	512	8

## Data Availability

The genomes of *Trichoderma harzianum* M10 v1.0 (https://genome.jgi.doe.gov/portal/TriharMinimDraft_2_FD/TriharMinimDraft_2_FD.download.html (accessed on 11 May 2023)), *Trichoderma harzianum* T22 v1.0 (https://genome.jgi.doe.gov/portal/TriharT22_1/TriharT22_1.download.html (accessed on 11 May 2023)), *Trichoderma harzianum TR274 v1.0* (https://genome.jgi.doe.gov/portal/Trihar1/Trihar1.download.html (accessed on 11 May 2023)) *and Trichoderma harzianum CBS 226.95 v1.0* (https://genome.jgi.doe.gov/portal/Triha1/Triha1.download.html (accessed on 11 May 2023)) were downloaded from the JGI Genome Portal with permission from the authors. Sequences of all analyses performed in the present study were deposited in repository on https://github.com/Suhadbio (accessed on 11 May 2023).

## Supplementary Materials

The following supporting information can be downloaded at: https://www.mdpi.com/article/10.3390/jof9090895/s1, Table S1: *Trichoderma* species and their predicted SMs core enzymes and their associated BGCs; Table S2: Tricho-NRPS1 Predicted BGC; Table S3: Tricho-NRPS2 Predicted BGC; Table S4: Tricho-NRPS3 Predicted BGC; Table S5: Tricho-NRPS4 Predicted BGC; Table S6: Tricho-NRPS5 Predicted BGC; Table S7: Tricho-NRPS7 Predicted BGC; Table S8: Tricho-NRPS8 Predicted BGC; Table S9: Tricho-NRPS11 Predicted BGC; Table S10: Tricho-NRPS12 Predicted BGC; Table S11: Tricho-NRPS13 Predicted BGC; Table S12: Tricho-NRPS14 Predicted BGC; Table S13: Tricho-NRPS15 Predicted BGC; Table S14: Tricho-NRPS-Like2 Predicted BGC; Table S15: Tricho-NRPS-Like4 Predicted BGC; Table S16: Tricho-NRPS-Like5 Predicted BGC; Table S17: Tricho-NRPS-Like6 Predicted BGC; Table S18: Tricho-NRPS-Like7 Predicted BGC; Table S19: Tricho-NRPS-Like8 Predicted BGC; Table S20: Tricho-NRPS-Like9 Predicted BGC; Table S21: Tricho-PKS1 Predicted BGC; Table S22: Tricho-PKS2 Predicted BGC; Table S23: Tricho-PKS3 Predicted BGC; Table S24: Tricho-PKS4 Predicted BGC; Table S25: Tricho-PKS5 Predicted BGC; Table S26: Tricho-PKS6 Predicted BGC; Table S27: Tricho-PKS7 Predicted BGC; Table S28: Tricho-PKS8 Predicted BGC; Table S29: Tricho-PKS9 Predicted BGC; Table S30: Tricho-PKS10 Predicted BGC; Table S31: Tricho-PKS11 Predicted BGC; Table S32: Tricho-PKS12 Predicted BGC; Table S33: Tricho-PKS13 Predicted BGC; Table S34: Tricho-PKS15 Predicted BGC; Table S35: Tricho-PKS17 Predicted BGC; Table S36: Tricho-PKS18 Predicted BGC; Table S37: Tricho-PKS19 Predicted BGC; Table S38: Tricho-PKS20 Predicted BGC; Table S39: Tricho-PKS like1 Predicted BGC; Table S40: Tricho-PKS like2 Predicted BGC; Table S41: Tricho-PKS like3 Predicted BGC; Table S42: Tricho-HrPKS1 Predicted BGC; Table S43: Tricho-HrPKS2 Predicted BGC; Table S44: Tricho-HrPKS3 Predicted BGC; Table S45: Tricho-HrPKS4 Predicted BGC; Table S46: Tricho-HrPKS5 Predicted BGC; Table S47: Tricho-HrPKS6 Predicted BGC; Table S48: Tricho-TC1 Predicted BGC; Table S49: Tricho-TC2 Predicted BGC; Table S50: Tricho-TC3 Predicted BGC; Table S51: Tricho-TC4 Predicted BGC; Table S52: Tricho-TC5 Predicted BGC; Table S53: Tricho-DMAT Predicted BGC.
