# Supplementary material for: Genomic Based Analysis of the Biocontrol Species Trichoderma harzianum: A Model Resource of Structurally Diverse Pharmaceuticals and Biopesticides
Source: J Fungi (Basel). 2023 Aug 31;9(9):895. doi: 10.3390/jof9090895 (PMC10532697; doi:10.3390/jof9090895)
Supplement: Supplementary file 1 [file jof-09-00895-s001.zip › jof-2488993-supplementary.pdf]

Journal of *Fungi*

MDPI

Genomic Based Analysis of The Biocontrol Species *Trichoderma harzianum*: A Model Resource of Structurally Diverse Pharmaceuticals and Biopesticides

Supplementary information

List of tables

Table S1 represents the predicted core enzymes of the SMs of eighty-seven Trichoderma species genomes.

Tables from S1 to S20 represent predicted BGC for Tricho-NRPS

Tables from S21 to S38 represent predicted BGC for Tricho-PKS

Tables from S38 to S40 represent predicted BGC for Tricho-PKS like

Tables from S41 to S46 represent predicted BGC for Tricho-HrPKS

Tables from S47 to S51 represent predicted BGC for Tricho-TC

Table S52 represents predicted BGC for Tricho-DMAT

Table S1. *Trichoderma* species and their predicted SMs core enzymes and their associated BGCs.

	Trichoderma species	DMAT clusters	Hybrid clusters	NRPS clusters	NRPS like clusters	PKS clusters	PKS like clusters	TC clusters	Total core enzymes	Total BGCs
1	Trichoderma arundinaceum IBT 40837	0	4	13	9	22	3	4	55	33
2	Trichoderma asperelloides T203 v1.0	0	1	10	12	13	3	5	45	35
3	Trichoderma asperelloides TR356 v1.0	0	3	11	12	12	3	5	46	35
4	Trichoderma asperellum CBS 433.97 v1.0	0	2	13	11	12	2	4	44	34
5	Trichoderma atrobrunneum ITEM 908	0	2	4	7	17	2	4	36	20
6	Trichoderma atroviride v2.0	0	2	9	10	13	2	3	39	32
7	Trichoderma brevicompactum IBT40841	1	3	18	9	27	3	6	67	43
8	Trichoderma citrinoviride TUCIM 6016 v4.0	0	2	8	7	10	2	4	33	24
9	Trichoderma gamsii T6085	0	1	10	11	13	2	2	39	26
10	Trichoderma guizhouense NJAU 4742	0	6	12	8	20	2	6	54	41
11	Trichoderma hamatum GD12	0	2	13	11	15	2	3	46	24
12	Trichoderma harzianum CBS 226.95 v1.0	0	5	13	10	21	2	6	57	52
13	Trichoderma harzianum M10 v1.0	1	6	15	10	20	3	6	61	51
14	Trichoderma harzianum T22 v1.0	0	6	15	10	19	3	6	59	48

15	Trichoderma harzianum TR274 v1.0	0	5	14	9	20	2	6	56	41
16	Trichoderma longibrachiatum ATCC 18648 v3.0	0	1	7	5	9	3	3	28	22
17	Trichoderma parareesei CBS 125925	0	1	7	6	10	2	4	30	16
18	Trichoderma pleuroti TPhu1	0	4	13	11	21	3	8	58	40
19	Trichoderma reesei CBS999.97 MAT1-1	0	1	8	6	9	2	4	30	23
20	Trichoderma reesei CBS999.97 MAT1-2	0	1	7	7	8	2	4	29	22
21	Trichoderma reesei QM6a	0	1	8	5	10	2	4	30	19
22	Trichoderma reesei RUT C-30 v1.0	0	2	8	5	8	2	4	29	21
23	Trichoderma reesei v2.0	0	2	8	5	9	2	4	30	20

Table S2. Tricho-NRPS1 Predicted BGC.

**Tricho-NRPS1 Predicted BGC**							
**Protein ID**	**1**	**2**	**3**	**4**	**5**	**6**	**7**
	**515467**	**476182**	**412883**	**505529**	**505532**	**515469**	**439904**
Function	Short chain dehydrogenase	N-terminal domain	Transcription factor	Alcohol dehydrogenase	PKS with 6 domains	TAP like protein	Phosphotransferase

Table S3. Tricho-NRPS2 Predicted BGC.

**Tricho-NRPS2 Predicted BGC**			
**Protein ID**	**1**	**2**	**3**
	**180300**	**180295**	**356455**
Function	Transcription factor	L-lysine 6 monooxygenase	AMP binding enzyme

Table S4. Tricho-NRPS3 Predicted BGC.

**Tricho-NRPS3 Predicted BGC**								
**Protein ID**	**1**	**2**	**3**	**4**	**5**	**6**	**7**	**8**
	**188187**	**188218**	**514122**	**474580**	**435587**	**474582**	**188396**	**451994**
Function	AMP-binding enzyme	Hypothetical protein	AMP-binding enzyme	Acetyltransferase	Major facilitator	Pyridine nucleotide disulphide oxidoreductase	Hypothetical protein	ABC transporter

Table S5. Tricho-NRPS4 Predicted BGC.

Tricho-NRPS4 Predicted BGC												
Protein ID	1	2	3	4	5	6	7	8	9	10	11	12
	351544	496372	496373	496375	351473	496384	351445	496401	449706	496410	472653	351441
Function	Pyridine nucleotide disulphide oxidoreductase	Cytochrome P450	O-methyltransferase	ABC transporter	Cytochrome P450	Hypothetical protein	Glutathione S-transferase	Membrane dipeptidase	Hypothetical protein	NRP with 4 domains	Cytochrome P450	aminotransferase

Table S6. Tricho-NRPS5 Predicted BGC.

Tricho-NRPS5 Predicted BGC							
Protein ID	1	2	3	4	5	6	7
	338536	418640	338363	495778	123919	495780	495783
Function	FAD binding	Aldo/keto reductase	Cytochrome P450	FAD binding	Transcription factor	Cytochrome P450	NRP with 3 domains

Table S7. Tricho-NRPS7 Predicted BGC.

	Tricho-NRPS7 Predicted BGC																
**Protein ID**	**1**	**2**	**3**	**4**	**5**	**6**	**7**	**8**	**9**	**10**	**11**	**12**	**13**	**14**	**15**	**16**	**17**
	**419706**	**173371**	**468462**	**340094**	**508977**	**500083**	**517847**	**478762**	**419717**	**468468**	**517850**	**500099**	**173458**	**173469**	**500108**	**173494**	**419727**
Function	ABC transporter	Hypothetical protein	Cytochrome P450	Cytochrome P450	Hypothetical protein	Indoleamine 2,3-dioxgenase	Hypothetical protein	NRP with 4 domains	Alpha-beta hydrolase	Major facilitator	Hypothetical protein	Hypothetical protein	Hypothetical protein	NAD binding domain	Transcription factor	Phytanoyl-Co-A dioxygenase	NAD dependent epimerase/dehydratase

Table S8. Tricho-NRPS8 Predicted BGC.

	Tricho-NRPS8 Predicted BGC				
Protein ID	1	2	3	4	5
	419041	498049	393322	508707	498051
Function	Major facilitator	Hypothetical protein	Polysaccharide biosynthesis	NRP with 4 domains	NRPS

Table S9. Tricho-NRPS11 Predicted BGC.

	Tricho-NRPS11 Predicted BGC					
Protein ID	1	2	3	4	5	6
	477104	35437	415197	489837	487119	489848

Function	Fungal transcription factor	SDS	Hypothetical protein	NRP with 4 domains	ABC transporter	AMP binding

Table S10. Tricho-NRPS12 Predicted BGC.

Tricho-NRPS12 Predicted BGC							
Protein ID	1	2	3	4	5	6	7
	504003	360465	360482	411624	465207	525012	321479
Function	Zn(2)Cys	ABC transporter	Peptide transporter	aminotransferase	Peptidase	NRP with 6 domains	Short chain dehydrogenase

Table S11. Tricho-NRPS13 Predicted BGC.

Tricho-NRPS13 Predicted BGC											
Protein ID	1	2	3	4	5	6	7	8	9	10	11
	502851	215460	385558	502872	385564	514734	464778	215503	437689	502879	475307
Function	Cytochrome P450	Transcription factor	Pyridine nucleotide disulphi	AMP binding enzyme	Hypothetical protein	ABC transporter	Enoly-CoA hydratase/isomerase	Major facilitator	Short chain dehydrogenase	Hypothetical protein	Major facilitator

			de oxidore ductase								

Table S12. Tricho-NRPS14 Predicted BGC.

Tricho-NRPS14 Predicted BGC					
Protein ID	1	2	3	4	5
	359352	410736	359428	524679	385620
Function	Amino acid permease	Transcription factor	Glycosyl hydrolase	Hypothetical protein	NRP with 3 domains

Table S13. Tricho-NRPS15 Predicted BGC.

Tricho-NRPS15 Predicted BGC									
Protein ID	1	2	3	4	5	6	7	8	9
	358358	436838	464438	502361	436848	436850	452487	514490	502372
Function	Transcrip tion factor	Phosphat ase catalytic	Hypothet ical protein	NRP with 4 domains	Hypothet ical protein	Hypothet ical protein	Cys/Met metabolis m	O-methyltra nsferase	Metallo-beta-lactamase

Table S14. Tricho-NRPS-Like2 Predicted BGC.

Tricho-NRPS-Like2 Predicted BGC												
Protein ID	1	2	3	4	5	6	7	8	9	10	11	12
	408559	451842	356753	474446	185283	356690	463816	383423	500937	435306	500939	500943
Function	Cytochrome P450	F-box like	Protein kinase domain	Aldo-keto reductase	Transcription factor	Major facilitator	NRPs-like with 4 domains	Glutathione S-transferase	Alpha/beta hydrolase	hypothetical	Sugar transporter	Zn(2)-Cys(6) binuclear

Table S15. Tricho-NRPS-Like4 Predicted BGC.

Tricho-NRPS-Like4 Predicted BGC								
Protein ID	1	2	3	4	5	6	7	8
	464044	409062	501348	524034	383932	501353	357428	501356
Function	FAD binding	Major facilitator	NRPs with 3 domains	aminotransferase	Arginase family	Hypothetical protein	Major facilitator	NAD dependent epimerase/dehydratase

Table S16. Tricho-NRPS-Like5 Predicted BGC.

Tricho-NRPS-Like5 Predicted BGC																					
Protein ID	1	2	3	4	5	6	7	8	9	10	11	12	13	14	15	16	17	18	19	20	21
	382256	434214	513631	434215	407409	382269	407415	434218	434219	499572	499574	499579	407425	434224	434225	451301	473985	434231	434233	499609	168848
Function	NADH(P) binding	Alpha-beta hydrolase	Aldo-keto reductase	Amidohydrolase	Hypothetical protein	Cytochrom b5	FAD binding domain	Fugal Zn(2)-Cys(6) binuclear	Major failitator	Major facilitator	NRPs like with 3 domains	Hypothetical protein	NmrA-like family	Major facilitator	NmrA-like family	Enoyl-Acyl carrier protein reductase	FR47-like protein	FAD binding	Pyoverdine protein	DNA repair metallo-beta-lactamase	Cytochrome P450

Table S17. Tricho-NRPS-Like6 Predicted BGC.

Tricho-NRPS-Like6 Predicted BGC												
Protein ID	1	2	3	4	5	6	7	8	9	10	11	12
	483855	406560	406563	498918	433222	354387	462878	406571	450870	161723	433238	433239
Function	Amino acid permease	Taurine catabolism dehydrogenase	Major facilitator	NRPs like with 4 domains	Fungal Zn(2)-Cys(6) binuclear	Glycosyl transferase	Sulfatase	Phosphoesterase	Glutathion S-transferase	Transcription factor	Phosphoribulokinase	Transcription factor

Table S18. Tricho-NRPS-Like7 Predicted BGC.

Tricho-NRPS-Like7 Predicted BGC					
Protein ID	1	2	3	4	5
	432635	405996	432638	522823	432644
Function	NRPS like with 5 domains	Arylsulfotransferase	Hypothetical protein	RNA polymerase	Fungal transcription factor

Table S19. Tricho-NRPS-Like8 Predicted BGC.

Tricho-NRPS-Like8 Predicted BGC													
Protein ID	1	2	3	4	5	6	7	8	9	10	11	12	13
	482042	521159	471688	98880	98861	98860	482048	376679	428608	99051	471696	448562	99018
Function	NAD dependent epimerase	Rgp1	Phosphotransferase	Fe(II) oxygenase super family	Amidohydrolase	DNA-J domain	Alpha/beta hydrolase	NRPS like with 4 domains	Amino methyltransferase	Fungal Zn(2)-Cys(6) binuclear	Major facilitator	NADH-flavin oxidoreductase	Amino acid permease

Table S20. Tricho-NRPS-Like9 Predicted BGC.

Tricho-NRPS-Like9 Predicted BGC													
Protein ID	1	2	3	4	5	6	7	8	9	10	11	12	13
	521230	100953	376845	349373	482117	460771	376849	402025	471766	402033	349104	101004	521239
Function	Short chain dehydrogenase	Hypothetical protein	Hypothetical protein	Hypothetical protein	Major facilitator	Alpha-beta hydrolase	Alcohol acetyltransferase	NRPS like with 5 domains	Major facilitator	MaoC dehydratase	CoA-ligase	Hypothetical protein	Pyridine nucleotide disulphide oxidoreductase

Table S21. Tricho-PKS1 Predicted BGC.

Tricho-PKS1 Predicted BGC		
Protein ID	1	2
	499215	499219
Function	AMP binding	PKS with 9 domains

Table S22. Tricho-PKS2 Predicted BGC.

Tricho-PKS2 Predicted BGC						
Protein ID	1	2	3	4	5	6
	164603	164607	164644	433750	164697	381909
Function	Fungal Zn(2)-Cys(6) binuclear	Glutathione-dpenedent formaldehyde-activity enzyme	Hypothetical protein	O-methyltransferase	Scylatone dehydratase	PKS with 5 domains

Table S23. Tricho-PKS3 Predicted BGC.

Tricho-PKS3 Predicted BGC										
Protein ID	1	2	3	4	5	6	7	8	9	10
	483114	431297	431298	496821	522268	136284	404601	404602	379445	512670
Function	O-methyltransferase	Methyltransferase domain	Alpha beta hydrolase	Short chain dehydrogenase	Fungal Zn(2)-Cys(6) binuclear	PKS with 5 domains	FAD binding domain	Thioesterase	PKS with 5 domains	ABC transporter

Table S24. Tricho-PKS4 Predicted BGC.

Tricho-PKS4 Predicted BGC						
Protein ID	1	2	3	4	5	6
	522072	404130	482934	430881	430885	522077
Function	Cytochrome P450	Lipase Acylhydrolase	PKS with 6 domains	Hypothetical protein	Pyridine nucleotide-disulphide	FAD binding protein

Table S25. Tricho-PKS5 Predicted BGC.

Tricho-PKS5 Predicted BGC													
Protein ID	1	2	3	4	5	6	7	8	9	10	11	12	13
	431418	462073	136943	431422	496982	404776	431425	431427	497001	522355	352294	431433	431434
Function	FAD binding	Methyltransferase	Major facilitator	Taurine catabolism dioxygenase	NmrA-Like family	Pyridoxal phosphate dependent enzyme	Major facilitator	Hypothetical protein	PKS with 7 domains	Multicopper oxidase	Fungal chitosanase of glycosyl hydrolase	GMC oxidoreductase	Major facilitator

Table S26. Tricho-PKS6 Predicted BGC.

Tricho-PKS6 Predicted BGC								
Protein ID	1	2	3	4	5	6	7	8
	442855	477362	390772	365481	48567	442869	442870	491174
Function	Major facilitator	Cytochrome P450	Serine hydrolase	PKS with 8 domains	Short chain dehydrogenase	Aflatoxin regulatory	FAD binding	PKS with 4 domains

Table S27. Tricho-PKS7 Predicted BGC.

Tricho-PKS7 Predicted BGC					
Protein ID	1	2	3	4	5
	443029	487562	336484	487566	391037
Function	DSS1/SEM family	Phosphoglycerate kinase	Mpv/PMP family	Hypothetical protein	PKS with 3 domains

Table S28. Tricho-PKS8 Predicted BGC.

Tricho-PKS8 Predicted BGC						
Protein ID	1	2	3	4	5	6
	78633	417363	417370	455782	455783	392064
Function	Short chain dehydrogenase	Hypothetical protein	Hypothetical protein	Cytochrome P450	Serine hydrolase	PKS with 9 domains

Table S29. Tricho-PKS9 Predicted BGC.

Tricho-PKS9 Predicted BGC					
Protein ID	1	2	3	4	5
	417581	417583	80976	392266	455895
Function	Cytochrome P450	Methyltransferase	PKS with 8 domains	Cytochrome P450	Series hydrolase

Table S30. Tricho-PKS10 Predicted BGC.

Tricho-PKS10 Predicted BGC																
Protein ID	1	2	3	4	5	6	7	8	9	10	11	12	13	14	15	16
	517516	456395	368040	339163	456398	444138	508690	508691	508692	339351	419016	393288	419019	456407	148593	419022
Function	Fungal Zn(2)-Cys(6) binuclear	Hypothetical protein	Major failitator	NAD dependent epimerase/dehydratase	Carboxylesterase	Carboxylesterase	FAD binding	Snoal-Like domain	PKS with 7 domains	Fungal specific transcription factor	Alcohol dehydrogenase	Major facilitator	Hypothetical protein	FAD binding	PKS with 7 domains	Transferase family

Table S31. Tricho-PKS11 Predicted BGC.

Tricho-PKS11 Predicted BGC												
Protein ID	1	2	3	4	5	6	7	8	9	10	11	12
	517695	498336	498339	393702	498346	444345	152317	419428	517699	152338	419430	498351
Function	NmrA-like family	Rhodanese-like domain	NADPH dependent reductase	Methyltransferase	Hypothetical protein	Pyridine nucleotide-disulphide	PKS with 7 domains	Cytochrome P450	Alcohol dehydrogenase	Short chain dehydrogenase	FAD binding	Enoyl-CoA hydratase

Table S32. Tricho-PKS12 Predicted BGC.

Tricho-PKS12 Predicted BGC											
Protein ID	1	2	3	4	5	6	7	8	9	10	11
	393773	393776	368547	508872	419484	498453	498459	468330	419489	419490	517733
Function	FAD binding	Fungal specific transcriptio n factor	NmrA-Like family	Fe(II) oxygenase superfamil y	Serine hydrolase	PKS with 6 domains	Metallo-beta-lactamase	Tetrapepti de repeat	Pyridine nucleotide-disulphide	Hypothetic al protein	Cytochrom e P450

Table S33. Tricho-PKS13 Predicted BGC.

Tricho-PKS13 Predicted BGC						
Protein ID	1	2	3	4	5	6
	255874	255870	469409	255932	255926	422265
Function	PKS with 8 domains	Glycosyltranferase	Intron endoulease	Taurine catabolism dioxygenase	SGC domain	Prenyltransferase and squalene oxidase

Table S34. Tricho-PKS15 Predicted BGC.

Tricho-PKS15 Predicted BGC		
Protein ID	1	2
	14131	479825
Function	O-methyltransferase	PKS with 5 domains

Table S35. Tricho-PKS17 Predicted BGC.

Tricho-PKS17 Predicted BGC													
Protein ID	1	2	3	4	5	6	7	8	9	10	11	12	13
	364464	489695	364451	34389	364477	487058	489706	389951	489715	389954	516189	442169	489737
Function	Short chain dehydrogenase	Hypothetical protein	Hypothetical protein	Fungal specific tanscription factor	NmrA-like family	Glutathion S-transferase	Amidase	PKS with 8 domains	Cytochrome P450	O-methyltransferase	Cytochrome P450	Cytochrome P450	Metallo-beta-lactamase

Table S36. Tricho-PKS18 Predicted BGC.

Tricho-PKS18 Predicted BGC		
Protein ID	1	2
	228413	411257
Function	Serine hydrolase	PKS with 7 domains

Table S37. Tricho-PKS19 Predicted BGC.

Tricho-PKS19 Predicted BGC														
Protein ID	1	2	3	4	5	6	7	8	9	10	11	12	13	14
	437247	464615	358912	475157	211896	211902	502627	475161	410349	502630	502631	358864	410352	358773
Function	Short chain dehydro genase	Fungal Zn(2)-Cys(6) binclear	Major facilitato r	Short chain dehydro genase	Short chain dehydro genase	tRNA syntheta ses	FAD binding	Cupin domain	Short chain dehydro genase	FAD binding	Cytochr ome P450	Short chain dehydro genase	Enoyl-Acyl carrier	PKS with 7 domains

Table S38. Tricho-PKS20 Predicted BGC.

Tricho-PKS20 Predicted BGC						
Protein ID	1	2	3	4	5	6
	502963	502967	502971	485477	437743	410755
Function	PKS with 5 domains	Hypothetical protein	Hypothetical protein	PKS with 4 domains	ABC transporter	ABC transporter

Table S39. Tricho-PKS like1 Predicted BGC.

Tricho-PKS like1 Predicted BGC										
Protein ID	1	2	3	4	5	6	7	8	9	10
	194678	436232	436236	384256	327515	357742	484956	194785	464192	357801
Function	Fungal specific transcription factor	Oligopeptide transporter	PKS with 2 domains	Ubiquinol-Cytochrome C	Dihydrofolate reductase	DSBA like thioredoxin	Short chain dehydrogenas e	ATPase family	Hypothetical protein	Fungal specific transcription factor

Table S40. Tricho-PKS like2 Predicted BGC.

Tricho-PKS like2 Predicted BGC									
Protein ID	1	2	3	4	5	6	7	8	9
	169707	451343	434338	463339	484249	434343	513680	169766	434346
Function	Fungal specific transcription factor	Hypothetical protein	Amidohydrolase	Acyltransferase	PKS with 4 domains	LSM domain	Pentatricopeptide repeat	Major facilitator	Fungal Zn(2)-Cys(6) binuclear cluster

Table S41. Tricho-PKS like3 Predicted BGC.

Tricho-PKS like3 Predicted BGC												
Protein ID	1	2	3	4	5	6	7	8	9	10	11	12
	517861	340247	339620	508989	173616	339971	419752	173658	173635	419758	517868	419761
Function	Serine hydrolase	Beta-ketoacyl synthase	Acyl transferase	PKS with 5 domains	Acetyltransferase	Hypothetical protein	Amino acid permease	Hypothetical protein	Fungal cellulose binding	Hypothetical protein	Carbon nitrogen hydrolase	Fungal specific transcription factor

Table S42. Tricho-HrPKS1 Predicted BGC.

Tricho-HrPKS1 Predicted BGC									
Protein ID	1	2	3	4	5	6	7	8	9
	435166	474382	435173	435175	356492	435179	183129	182809	451770

Function	major facilitator superfamily)	Transcription factor	Pyruvate/Phosphoenolpyruvate kinase	O-methyltransferase	Aminotransferase	Hypothetical protein	Pyruvate/Phosphoenolpyruvate kinase	Alcohol dehydrogenase	AMP-dependent synthetase

Table S43. Tricho-HrPKS2 Predicted BGC.

Tricho-HrPKS2 Predicted BGC								
Protein ID	1	2	3	4	5	6	7	8
	479203	420937	503349	509354	395107	369895	395113	369902
Function	AMP-dependent synthetase	Hypothetical protein	Hypothetical protein	FOG: Zn-finger	Short-chain dehydrogenase/reductase	Voltage-gated shaker-like K+	Zn(2)-C6 fungal-type DNA-binding domain	Hypothetical protein

Table S44. Tricho-HrPKS3 Predicted BGC.

Tricho-HrPKS3 Predicted BGC																		
Protein ID	1	2	3	4	5	6	7	8	9	10	11	12	13	14	15	16	17	18
	468797	204429	420683	420688	204542	468801	457203	394862	502071	468805	468806	394863	502078	502080	420699	502081	479111	340292
Function	Sugar transporter	Hypothetical protein	Multiple inositol polyp	Alpha-isopropylmalat	Monocarboxylate trans	Transcription factor	Hypothetical protein	AMP-dependent	Cytochrome P450	Monooxygenase	Alcohol dehy	Hypothetical protein	O-methyltransferase	Transcription factor	Hypothetical protein	FAD-binding	Alcohol dehydrogenase	Cytochrome P450

			hosp hate phos phata se	e synth ase	porte r			synth etase			droge nase							

Table S45. Tricho-HrPKS4 Predicted BGC.

Tricho-HrPKS4 Predicted BGC				
Protein ID	1	2	3	4
	480138	519257	489253	480140
Function	Alcohol dehydrogenas e	Kynurenine 3-monooxygena se	Iron/ascorbate family oxidoreductas es	AMP-dependent synthetase

Table S46. Tricho-HrPKS5 Predicted BGC.

Tricho-HrPKS5 Predicted BGC													
Protein ID	1	2	3	4	5	6	7	8	9	10	11	12	13
	45880 0	48048 2	49066 1	44661 7	42428 3	42428 4	34452 0	39819 9	34446 2	34451 3	47016 8	34453 5	398203
Function	Flavo nol	Short - chain	Gluta thion e-	Hypo thetic al	Hypo thetic al	Hypo thetic al	Aspa rtyl	AMP-depe ndent	Gluta thion e S-	Hypo thetic al	Major facilit ator	Alde hyde dehy	Cytochrome P450

	reductase	dehydrogenase	dependent formaldehyde-activating enzyme	protein	protein	protein	protease	synthetase	transferase	protein	superfamily	drogenase		

Table S47. Tricho-HrPKS6 Predicted BGC.

Tricho-HrPKS6 Predicted BGC						
Protein ID	1	2	3	4	5	6
	475760	504253	504256	475763	504262	360640
Function	Cytochrome P450	Acetylcholinesterase	Zn(2)-C6 fungal-type DNA-binding domain	Short-chain dehydrogenase	AMP-dependent synthetase	Alcohol dehydrogenase

Table S48. Tricho-TC1 Predicted BGC.

Tricho-TC1 Predicted BGC						
Protein ID	1	2	3	4	5	6
	500701	356522	500708	500710	500717	500722

Function	Glutathion S-transferase	Sugar transporter	Glycosyl hydrolase	Terpene synthase	Capsular polysaccharide synthesis	Cytochrome P450

Table S49. Tricho-TC2 Predicted BGC.

Tricho-TC2 Predicted BGC		
Protein ID	1	2
	514100	501120
Function	Cytochrome P450	Terpene synthase

Table S50. Tricho-TC3 Predicted BGC.

Tricho-TC3 Predicted BGC					
Protein ID	1	2	3	4	5
	406447	160738	433134	522997	354266
Function	NAD dependent epimerase/ dehydratase	Dienelactone hydrolase	Hypothetical protein	Hypothetical protein	Terpene synthase family

Table S51. Tricho-TC4 Predicted BGC.

Tricho-TC4 Predicted BGC										
Protein ID	1	2	3	4	5	6	7	8	9	10
	403973	403974	130100	351573	351479	512447	351344	130112	378819	482873
Function	Terpene synthase	Hypothetic al protein	FAD binding	Hypothetic al protein	Alpha-beta hydrolase	Major facilitator	NmrA-like family	WD,G-beta repeat	Hypothetic al protein	Glutathione S-transferase

Table S52. Tricho-TC5 Predicted BGC.

Tricho-TC5 Predicted BGC						
Protein ID	1	2	3	4	5	6
	424392	490911	490920	344624	490924	344808
Function	Metallo-beta-lactamase	Hypothetical protein	Hypothetical protein	Hypothetical protein	Hypothetical protein	Terpene synthase

Table S53. Tricho-DMAT Predicted BGC.

Tricho-DMAT Predicted BGC											
Protein ID	1	2	3	4	5	6	7	8	9	10	11
	57371	470720	57387	425630	399440	374262	425637	470726	425640	57476	425644
Function	Zn(2)-C6 fungal-type	Hypothe tical protein	Renal dipeptid ase	Major facilitato r	Methyltr ansferas e	Glutathi one S-	Cytochro me P450	Aromatic prenyltransf erase,	Aminocyc lopropane -1-	Non-ribosomal	Pyridine nucleotide-disulphide

	DNA-binding domain			superfamily		transferase		DMATS type	carboxylase synthase	peptide synthetase	oxidoreductase
